# Diallyl disulfide attenuated carbon ion irradiation-induced apoptosis in mouse testis through changing the ratio of Tap73/ΔNp73 via mitochondrial pathway

**DOI:** 10.1038/srep16020

**Published:** 2015-11-03

**Authors:** Cui-xia Di, Lu Han, Hong Zhang, Shuai Xu, Ai-hong Mao, Chao Sun, Yang Liu, Jing Si, Hong-yan Li, Xin Zhou, Bing Liu, Guo-ying Miao

**Affiliations:** 1 Department of Heavy Ion Radiation Medicine, Institute of Modern Physics, Chinese Academy of Sciences, Lanzhou 730000, China; 2 Key Laboratory of Heavy Ion Radiation Biology and Medicine of Chinese Academy of Sciences, Lanzhou 730000, China; 3 Key Laboratory of Heavy Ion Radiation Medicine of Gansu Province, Lanzhou 730000, China; 4 College of Stomatology, Lanzhou University, Lanzhou 730000, China

## Abstract

Diallyl disulfide (DADS), a major organosulfur compound derived from garlic, has various biological properties, including anti-cancer effects. However, the protective mechanism of DADS against radiation-induced mouse testis cell apoptosis has not been elucidated. In this study, the magnitude of radiation effects evoked by carbon ion irradiation was marked by morphology changes, significant rise in apoptotic cells, activation expression of p53, up regulation the ratio of pro-apoptotic Tap73/anti-apoptotic ΔNp73, as well as alterations of crucial mediator of the mitochondrial pathway. Interestingly, pretreatment with DADS attenuated carbon ion irradiation-induced morphology damages and apoptotic cells. Additionally, DADS elevated radiation-induced p53 and p21 expression, suggesting that p53 might be involved in the inhibition of cell cycle progression through up regulation of p21. Furthermore, administration with DADS prevented radiation-induced Tap73/ΔNp73 expression and consequently down regulated Bax/Bcl-2 ratio, cytochrome c release and caspase-3 expression, indicating that the balance between Tap73 and ΔNp73 had potential to activate p53 responsive genes. Thus, our results showed that radio protection effect of DADS on mouse testis is mediated by blocking apoptosis through changing the ratio of Tap73/ΔNp73 via mitochondrial pathway, suggesting that DADS could be used as a potential radio protection agent for the testis against heavy-ion radiation.

High- linear energy transfer (LET) heavy ions produce more cytotoxic and genotoxic damage to cells in comparison with low-LET ionizing irradiation, such as X-rays or γ-rays^1^,^2^,^3^,^4^. Our previous studies demonstrated that exposure to heavy ions such as carbon ions caused decrease in testicular weight and sthenospermia, which affects the testicular development and breeding activity of males^5^,^6^,^7^. The reason is that the organ systems with proliferating cells are very sensitive, and the testis is a radiosensitive organ. Additionally, in space travel, astronaut exposed to high-LET galactic cosmanic rays at higher radiation doses and dose rates than humans received on Earth. It is an unavoidable problem for long duration manned interplanetary missions^8^,^9^. Furthermore, in hadrontherapy, although high-LET heavy ions as an innovative modality of high precision tool for the treatment of cancer such as testis and prostate tumor, potential damage to healthy tissues surrounding the tumor target along its penetrating path should still be considered^10^. Given space radiation protection and cancer therapy, there is a demand for reliable agent for protection of the testis against heavy-ion radiation.

Recently, significant attention has been focused on natural radio protective agent in vegetables and fruits. Garlic (Allium sativum L.) is a popular spice added to several edible preparations all over the world and is a remedy for a variety of ailments. Diallyl disulfide (DADS; ie, CH2=CH-CH2-S-S-CH2-CH=CH2), an oil-soluble compound extracted from garlic documents as a potent compound to prevent cancer, genotoxicity, nephrotoxicity, and hepatotoxicity^11^,^12^,^13^,^14^,^15^,^16^. Previous studies have shown that DADS is not only effective at modulating apoptosis proteins but also has potent antiapoptosis capacity^12^. For example, a growing body of evidence indicates that DADS inhibits prostate, lung, gastric and breast cancer progression by inducing apoptosis^12^,^17^,^18^. Interestingly, a few studies have reported that pretreatment with DADS attenuated EtOH-induced gastric apoptotic changes, carbon tetrachloride-induced hepatic apoptotic changes, cyclophosphamide-induced urinary bladder epithelial cell apoptosis, and oxidative stress-injured neuronally differentiated PC12 cells, as evidenced by inhibition of cytochrome c release and caspase-3 activation^19^,^20^,^21^,^22^,^23^. It is well known that apoptosis is recognized as a key event in radiation damage and a determining factor of radiosensitivity^24^. However, the molecular mechanism of protective effect of DADS on carbon ion irradiation remains unclear. In this regard, based on ground experiments at accelerators, this study has been undertaken to estimate the protective mechanisms of DADS against carbon ion-induced cell apoptosis at the level of signal transduction pathway in mouse testis. We showed that DADS supplementation was better able to ameliorate radiation-induced morphological damage and apoptosis in mouse testis through regulating the ratio of Tap73/ΔNp73 via mitochondrial pathway, but not p53. These findings suggest that DADS is a very promising candidate for protection of the testis against heavy-ion radiation.

## Materials and Methods

### Ethics statement

Procedures were carried out in accordance with the Guidelines for Laboratory Animal Care. The experimental protocol was approved by the Ethical Committee of Institute of Modern Physics, Chinese Academy of Sciences, Lanzhou, China.

### Animals and reagents

**Figure 1:**
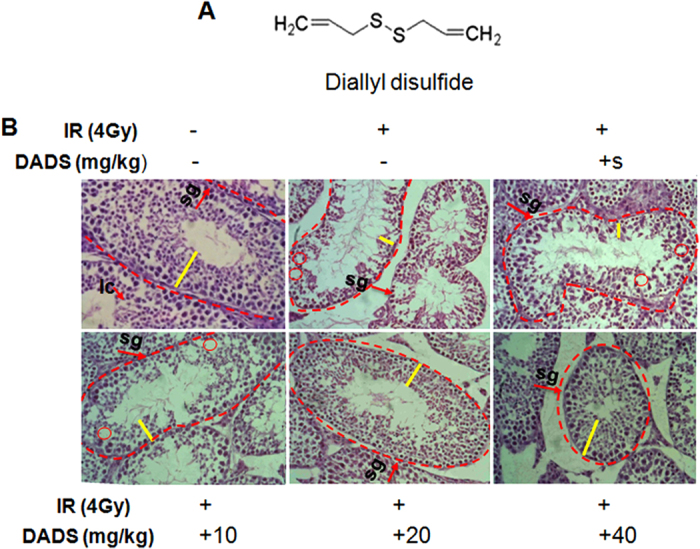
DADS significantly attenuates radiation-induced morphological damage in mouse testis. (**A**) Chemical structure of DADS. (**B**) Representative HE-stained histological sections of mouse testis treated with DADS (10 mg/kg, 20 mg/kg, and 40 mg/kg) and/or 4 Gy carbon ion irradiation. Seminiferous epithelium was thinner (yellow line) and cavities were formed in the seminiferous epithelium (dotted line circle) leading to hollow seminiferous tubules. Spermatogenic cells were disarranged and the spermatogonia should be close to the basement membrane. The basement membranes (arrow) were destroyed and disrupted (bracket). HE staining was performed at 40× magnification.

Young male mice (20 ± 2 g) of outbred Kun-Ming strain obtained from Lanzhou Medical College (Lanzhou, China) were used. All animal studies were carried out according to the requirements of the Animal Care Committee at the Institute. Mice were kept at a constant temperature (22 ± 1 °C) with 12 h light and dark cycles. DADS (C_6_H_10_S_2_, MW: 146.28, shown in Fig. 1A) was purchased from Sigma-Aldrich (St. Louis., MO, USA).

### Irradiation procedure

Whole-body irradiation of mice was performed using a high-LET carbon ion beams at the initial energy of 270 MeV/u and the average LET of 31.3 keV/μm in the water (the plateau region) generated from Heavy Ion Research Facility in Lanzhou (HIRFL, Institute of Modern Physics, Chinese Academy of Sciences, Lanzhou, China). Each mouse received 4 Gy dose at a dose rate of about 0.5 Gy/min. The collimation of the beams to the place irradiated was controlled by a microcomputer. The acquisition of data (preset numbers converted by doses of irradiation) was automatically accomplished using a microcomputer during irradiation. Particle fluence was determined from air ionizing chamber signal according to the calibration of the detector. Dose was calculated from particle fluence and LET.

### Sample collection

Animals were randomly divided into six groups each containing 10 individuals. The first group was just administered physiological saline solution (0.85% NaCl) only for 5 days as a control group. The second group of animals received only carbon ion irradiation as the irradiated group. The third group was injected intraperitoneally with peanut oil for 3 days before irradiation. The forth group was injected intraperitoneally with DADS at 10 mg/kg dissolved in peanut oil for 3 days before irradiation. The fifth group was treated with DADS at 20 mg/kg and the last group received DADS at 40 mg/kg for 3 days prior to carbon ion irradiation. The maximum effective dosage of DADS used in the present study was selected based on data from previous studies^20^,^23^,^25^ and intraperitoneal administration of 40 mg/kg melatonin alone for 5 days also did not significantly induce apoptotic cells with respect to the control group (data not shown), indicating that chronic treatment with 40 mg/kg DADS did not cause the toxicity effects to trigger apoptosis. After this, the animals were sacrificed by cervical dislocation 48 h after irradiation. Testis were quickly removed, and then fresh samples were immersed in 4% formaldehyde–phosphate buffer solution for hematoxylin and eosin staining (HE), TUNEL and immunofluorescence analysis. Residual samples were immediately frozen and stored at −80 °C until other biochemical determinations were carried out.

### Histopathological analysis

Mice were killed by cervical dislocation. The testis of each mouse was taken out. The fat and onnective tissues adhering to testis were removed. For histological analysis tissue specimens were fixed for 24 h in buffered formaldehyde solution (3.7% in PBS) at room temperature, dehydrated by graded ethanol and embedded in paraffin. Tissue sections (thickness: 5 μm) were deparaffinized with xylene and stained with HE. Digital images were captured and analyzed with a Nikon Eclipse TE2000-S microscope (magnification, 40×).

### Apoptosis detection

*In situ* cell apoptosis was evaluated by terminal deoxynucleotidyl transferase (TdT)-mediated dUTP nick end labeling (TUNEL) kit (Roche, Mannheim, Germany) as specified by the manufacturer’s protocol. Briefly, paraffin sections were deparaffined with xylene and rehydrated in a graded series of ethanol. Then the slides were washed and permeabilized by 5 min microwave irradiation (350 W) in 0.1 M itrate buffer (pH 6.0), and then incubated with blocking solution (0.1 M Tris–HCl, pH 7.5, and 3% BSA) for 30 min. Afterwards, the label solution (labeled nucleotides and TdT enzyme) was added for 60 min at 37 °C in the dark. Samples were rinsed, dried, and incubated with converter-POD for 30 min at 37 °C. Finally, sections were visualized using DAB (DAKO, Carpenteria, CA, USA). Only heavily stained cells were considered to be apoptotic.

### qRT-PCR

Quantitative real-time polymerase chain reaction (qRT-PCR) was performed using SYBR Green (Takara, Tokyo, Japan). Total RNA was isolated from mouse testis using Trizol Reagent (Invitrogen Life Technologies, Inc, CA, USA). qRT-PCR was performed using a Mx3000P real-time PCR System (Stratagene, La Jolla, CA, USA) with two-stage program parameters as follows: 2 min at 50 °C, 10 min at 95 °C, 40 cycles of 15 s at 95 °C and 1 min at 60 °C. Each sample was tested in triplicate, and the samples obtained from three independent experiments were used for the analysis of relative gene expression using the 2-∆∆ CT method. The primers were used for qRT-PCR as shown in table 1.

### Western blot analysis

Testicular tissue was homogenized in 1 ml lyses buffer containing 7 M urea, 2 M thiourea, 4% (w/v) 3-[(3-cholamidopropyl)-dimethylammonio]-1-propane sul-fonate (CHAPS), and 2% (w/v) dithiothreitol (DTT) in the presence of 1% (v/w) protease inhibitor cocktail (Sigma Chemical, St. Louis, MO, USA). The supernatant was collected, and the amount of protein was estimated by BCA protein assay kit. Total protein was electrophoresed on a reducing SDS polyacrylamide gel under standard conditions and electroblotted to PVDF membranes with 15% methanol, 25 mM Tris, and 192 mM glycine. Equal protein loading per lane was ensured by using an anti-β-Actin antibody. The membranes were blocked with 5% nonfat dry milk in TBS (10 mM Tris, pH 7.4, 100 mM NaCl) containing 0.01% Tween 20 for 1 h at room temperature and incubated with primary antibody (1:500 dilution for caspase-3, 1:1000 dilution for antibodies against p53, Bcl-2, Bax, p21, Tap73, ΔNp73, and cytochrome c; 1:10 000 dilution for β-Actin in 1% nonfat dry milk in TBS) overnight at 4 °C. After thorough washing, the membranes were incubated with secondary antibodies conjugated with horseradish peroxidase (1:5000 dilution for anti-rabbit and 1:10 000 dilution for anti-mouse antibody in 5% nonfat dry milk in TBS) and streptavidin-horseradish peroxidase (1:2500) for 1 h at room temperature. Secondary probes were detected by ECL western blot detection reagents (GE Healthcare, Piscataway, NJ, USA). The expression of protein was quantified using Fluor Chem FC2 software (Alpha Innotech Corporation, San Leandro, CA, USA). Protein expression was visualized using a standard chemiluminescence system.

### Immunofluorescence

The fixed tissues were thoroughly washed in 0.01 M phosphate buffer (pH 7.4), dehydrated in graded ethanol, toluene-cleared, and embedded in paraffin. A paraffin section of testis was cut at 5 μm, mounted on slides. Fixed sections were soaked in 3% H_2_O_2_ and incubated for 30 min; 1% Triton X-100 for 30 min; blocked for 25 min at room temperature by the drop wise addition of 5% BSA, primary antibody (1:500) 4 °C overnight; then incubated donkey anti-rabbit IgG (1:100) secondary antibody Texas red and FITC (Invitrogen Life Technologies, Inc, CA, USA) at 37 °C for 1 h; the above steps were repeated with 0. 01 M (pH 7.2–7.4) PBS and washed twice for 5 min each wash, then VECTASHIELD mounting medium with 4′,6-diamidino-2-phenylindole (DAPI) (Vector, Laboratories, USA) was added. A confocal laser microscope (LSM, Carl Zeiss AG, Germany) was used with the camera section on a Texas red (ΔNp73) and FITC green (Tap73) fluorescence was positive.

### Statistical analysis

Each experiment was repeated at least three times. Data are presented as the mean ± SD. Statistical analysis was performed by the Student’s t-test. A P value < 0.05 was selected as a criterion for a statistically significant difference.

## Results

### DADS attenuates radiation-induced morphological damage

Histological sections of control mouse showed regular-shaped seminiferous tubules, intact base mentmembrane, and regular, tight layers of spermatogenic cells (Fig. 1B). 48 h after carbon ion irradiation, it was showing radiation-induced pathological changes in irradiated group, in the form of disordered and shrunk seminiferous tubules, thinning seminiferous epithelium with loosely arranged cells, cavity formations, disarranged spermatogenic cells, and disrupted basement membrane were observed (Fig. 1B). Histological sections of mouse testis exposed to the irradiation showed drastic pathological lesions in tubular architecture when compared with control mice. However, DADS pretreatment rendered the quality as evident in the form of intact germinal epithelium, mild cytoplasmic vacuolization with the absence of karyolysis, pyknosis, and necrosis as well as increased germ cells number with a dosage- dependent manner (Fig. 1B).

### DADS alleviates radiation-induced apoptosis cell

**Figure 2:**
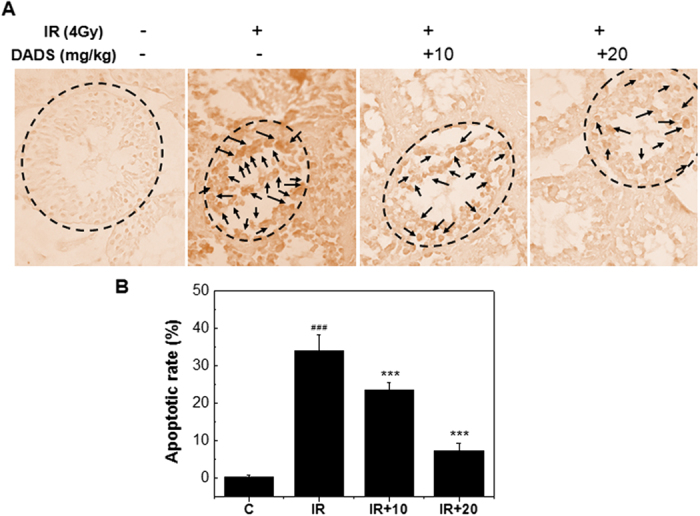
TUNEL stained histology of mouse testis sections at 48 h after carbon ion irradiation with or without DADS (Original magnification: 40X). (**A**) Representative TUNEL-stained sections of mouse testis treated with DADS (10 mg/kg, and 20 mg/kg) and/or 4 Gy carbon ion irradiation. (**B**) Apoptotic rate is apoptotic cells in randomly chosen histological fields. Each value is expressed as mean ± SD at least three independent experiments.

Apoptotic testis cells were detected by TUNEL staining. A large number of TUNEL-positive nuclei were observed in the testis tissues obtained 48 h after carbon ion irradiation (Fig. 2). Figure 2 showed that different concentrations of DADS pretreatment provided a marked reduction in the elevation of apoptotic testis cells induced by radiation, and there was a significant dose dependent decrease in irradiation-induced apoptotic cells with increasing DADS dosages. In particular, the apoptotic cells in 20 mg/kg dose of DADS-treated group was decreased significantly relative to the irradiated group (Fig. 2B).

### DADS up- regulates p53 and p21 expression in mouse testis exposed to carbon ion irradiation, indicating activation of DNA repair

**Figure 3:**
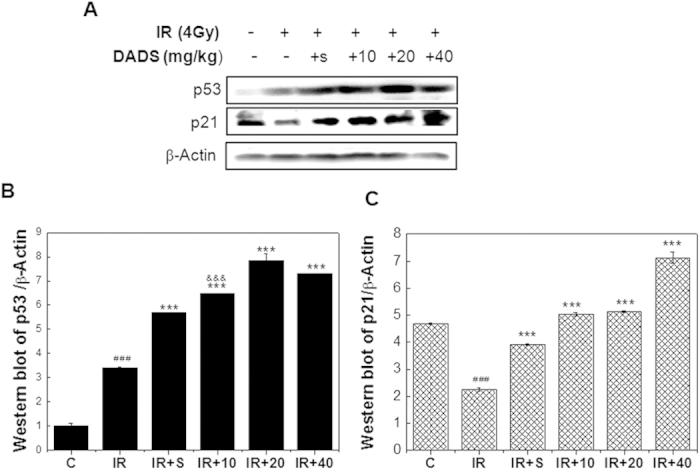
Effect of DADS on the protein levels of p53 and p21 in carbon ion–irradiated mouse testis. (**A**) Representative western blot images. (**B**,**C**) Quantitative analysis of p53 and p21 protein in mouse testis tissues by western blot analysis. β-Actine was used as a loading control. Relative expression of different protein compaired with control in the same time. Values represent the average ± SD from three gels per group. ^###^P < 0.001 versus the control group for IR group; ***P < 0.001 versus the IR group for the group received DADS for 3 days prior to carbon ion irradiation.

The tumor-suppressor protein p53 is responsible for many forms of genotoxic agent-induced apoptosis. In an attempt to find the molecular mechanism underlying DADS-reduced apoptosis, we investigated the role of p53 in mouse testis treated by DADS before and exposed to irradiation (Fig. 3). Western blot analysis showed that carbon ion irradiation increased protein levels of p53. Notably, when carbon ion-exposed mouse testis was pretreated with DADS, there was still rise in p53 level. p53 expression in carbon ion-exposed mice pretreated with DADS at 10, 20 and 40 mg/kg doses was respectively higher 1.9-fold, 2.3-fold and 2.2-fold than that of carbon ion-exposed group. In addition, as shown in Fig. 3, down regulation of p21 was observed in the mouse testis at 48 h after exposure to carbon ion particles, while DADS pretreatment obviously prevented the irradiation-induced decrease in p21. There are approximate 2.2, 2.3 and 3.2-fold inductions in the p21 expression in irradiation group pretreated with 10, 20 and 40 mg/kg DADS, respectively, in comparison with irradiation group. These results suggested that p53 might be involved in the inhibition of cell cycle progression through up regulation of p21 but does not appear to be critical for DADS-reduced apoptosis in mouse testis exposed to carbon ion irradiation.

### DADS inhibits the ratio of Tap73/ΔNp73 in mouse testis exposed to carbon ion irradiation, indicating inactivation of apoptosis

**Figure 4:**
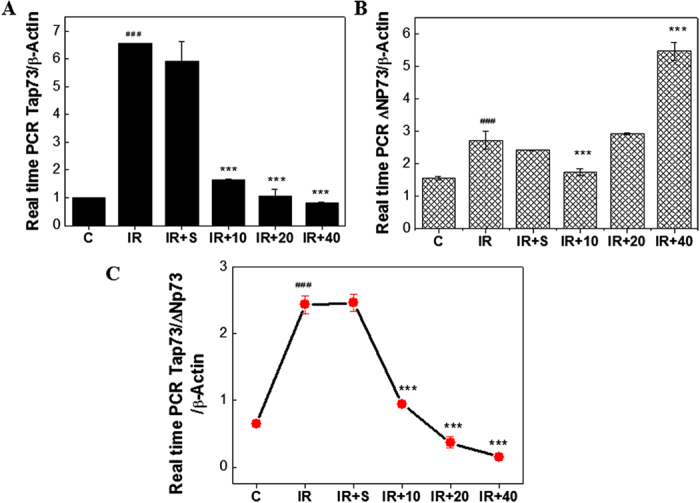
Effect of DADS on the mRNA levels of Tap73 and ΔNp73 in carbon ion–irradiated mouse testis. (**A**,**B**) The mouse testis supplemented with DADS was exposed 4 Gy carbon ion irradiation for 48 h and the expression of Tap73 and ΔNp73 mRNA were determined by qRT-PCR and normalized to β-Actin expression. (**C**) The relative levels of Tap73 and ΔNp73 mRNA are represented by dot graph. Relative expression of different gene compaired with control in the same time. Values are mean ± SD of at least three independent experiments.

Since p73 has been reported to play a crucial role in inducing apoptosis in response to radiation, in a p53-independent manner^26^,^27^,^28^, we examined if p73 is involved in DADS-reduced apoptosis induced by carbon ion irradiation. Real time PCR, western blot and immunofluorescence were applied to analyze the gene and protein expression, respectively. Tap73 and ΔNp73 mRNA transcript levels from each promoter of p73 were shown in Fig. 4. Levels of Tap73 mRNA were strongly increased in mouse testis at 48 h after 4 Gy carbon ion particles compared with control group. In contrast, levels of ΔNp73 mRNA were slowly increased. Interestingly, there was irradiation with carbon ions induced a sharp increase in the Tap73/ΔNp73 ratio, while DADS pretreatment obviously prevented this induction (Fig. 4C).

**Figure 5:**
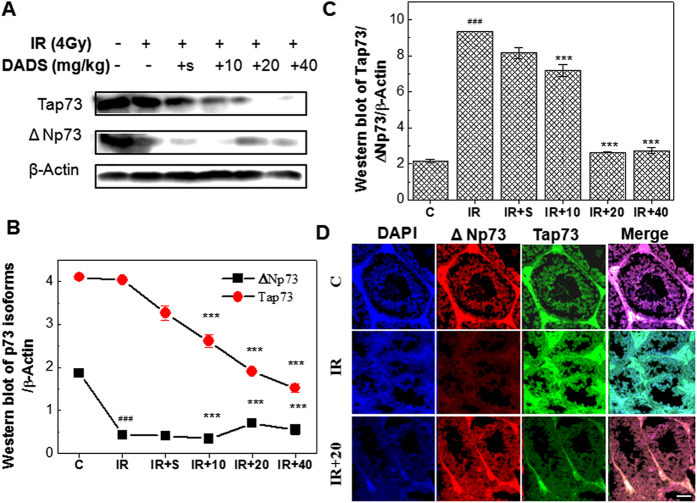
DADS inhibits the protein ratio of Tap73/ΔNp73 in mouse testis exposed to carbon ion irradiation, indicating inactivation of apoptosis. (**A**) Representative western blot images. (**B**,**C**) Quantitative analysis showed the carbon ion irradiation induced the changes of Tap73 and ΔNp73, and the protein ratio of Tap73/ΔNp73 in mouse testis, and this effect was blocked by DADS. (**D**) The mouse testis supplemented with DADS was exposed 4 Gy carbon ion irradiation for 48 h and the localization of Tap73 and ΔNp73 protein were determined by fluorescent microscopy and compared with normal mouse testis and mouse testis exposed to 4 Gy carbon ion irradiation for 48 h. Relative expression of different protein compaired with control in the same time. Values represent the average ± SD from three gels per group. ^###^P < 0.001 versus the control group for IR group; **P < 0.01 and ***P < 0.001 versus the irradiation group for the DADS + irradiation-treated groups.

We next examined the levels of Tap73 and ΔNp73 proteins by western blot analysis, using β-Actin as a control. As shown in Fig. 5 A, B, carbon ion irradiation induced a sharp decrease in ΔNp73 expression, and did not have much effect on Tap73 expression. It should be noted that this was not completely reflective of mRNA levels. However, carbon ion irradiation induced a sharp increase in the Tap73/ΔNp73 ratio, which was consistent with mRNA levels. In addition, the effect of DADS treatment on the protein level of Tap73/ΔNp73 ratio was in accordance with observed Tap73/ΔNp73 mRNA levels (Fig. 5 C).

To verify the expression of Tap73 and ΔNp73 proteins and examine the subcellular localization of these proteins, we next performed immunofluorescent staining. As shown in Fig. 5D, we detected Tap73 and ΔNp73 expression in the nucleus of mouse testis. Consistent with the western blot analysis, Tap73 expression was not obviously changed in mouse testis exposed to carbon ion irradiation compared with untreated mouse testis, while ΔNp73 expression was significantly decreased. Moreover, Consistent with the western blot analysis, DADS down- regulated Tap73 and up-regulated ΔNp73 expression in mouse testis at 48 h after exposure to carbon ion particles.

### DADS down-regulates Bax/Bcl-2 ratio, and prevents cytochrome c release and caspase activation in mouse testis exposed to carbon ion irradiation

**Figure 6:**
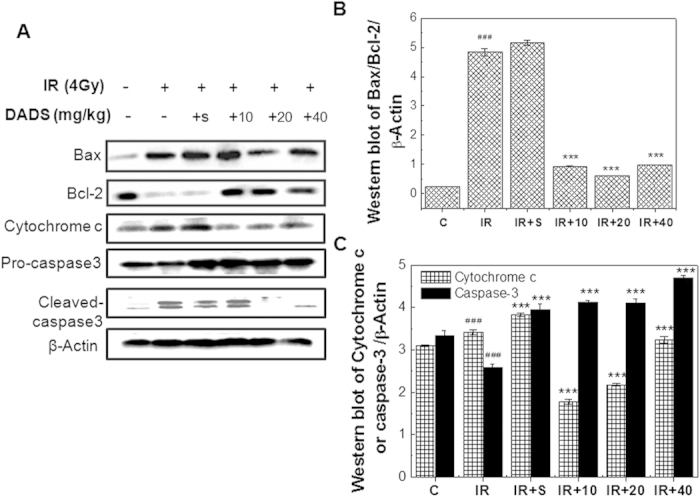
DADS down-regulates Bax/Bcl-2 ratio, and prevents cytochrome c release and caspase activation in mouse testis exposed to carbon ion irradiation. (**A**) Representative western blot images. (**B**) The ratio of Bax/Bcl-2 is represented by column graphs. (**B**,**C**) Quantitative analysis of the ratio of Bax/Bcl-2, cytochrome c, pro-caspase 3 and cleaved caspase 3 are represented by column graphs. β-Actine was used as a loading control. Relative expression of different protein compaired with control in the same time. Values represent the average ± SD from three gels per group. ^###^P < 0.001 versus the control group for IR group; **P < 0.01 and ***P < 0.001 versus the irradiation group for the DADS + irradiation-treated groups.

Bcl-2 family members, cytochrome c, and caspase-3 are crucial mediators of mitochondrial pathway of apoptosis. To determine whether DADS demote apoptosis via mitochondrial pathway in mouse testis exposed to carbon ion irradiation, we analyzed the expression of these mediators. As shown in Fig. 6, there was irradiation with carbon ions induced a sharp increase in the protein level of pro-apoptotic Bax and a sharp decrease in the protein level of anti-apoptotic Bcl-2, while DADS pretreatment obviously prevented the irradiation-induced increase in the Bax/Bcl-2 ratio. There are approximate 5.2, 8.0 and 4.9-fold reductions in the ratio of Bax/Bcl-2 expression in irradiation group pretreated with 10, 20 and 40 mg/kg DADS, respectively, in comparison with irradiation group (Fig. 6B). Additionally, the cytochrome c expression levels were upregulated at 48 h after carbon ion irradiation (P < 0.05). This elevation was prevented by the DADS treatment (Fig. 6). Furthermore, as shown in Fig. 6C, down regulation of caspase 3 was observed in the mouse testis at 48 h after exposure to carbon ion particles by appearance of the 35-kDa product and its cleaved form of 17 and 19 –kDa, while the DADS-pretreated samples showed up regulated expression of the corresponding caspase 3 and down regulated expression of its cleavage. These results indicated that DADS prevented radiation-induced apoptosis through mitochondrial pathway via down-regulating Bax/Bcl-2 ratio, cytochrome c release and caspase-3 levels.

## Discussions

Recently, significant attention has been focused on natural radio protective agent in vegetables and fruits. DADS, an oil-soluble compound, extracted from garlic documents as a potent compound to inhibit prostate, lung, gastric and breast cancer progression by inducing apoptosis^12^,^17^,^18^,^29^,^30^, but the molecular mechanism of protective effect of DADS on carbon ion irradiation remains unclear. In this regard, this study has been undertaken to estimate the protective mechanisms of DADS against carbon ion-induced cell apoptosis at the level of signal transduction pathway in mouse testis.

In the present study, the magnitude of radiation effects evoked by carbon ion irradiation was marked by morphology changes and activation of apoptosis. However, the DADS treatment showed a protective effect against the testicular toxicity induced by carbon ion irradiation in mouse, as evidenced by significantly attenuated radiation-induced morphological damage and decreased apoptotic cells in mouse testis. Kim *et al.* also reported that DADS pretreatment effectively attenuated the bladder toxicity caused by cyclophosphamide^31^. Thus, it is indicated that DADS has a potential to protect the testis against heavy-ion radiation.

The protective effect of DADS has been frequently attributed to p53. Numerous studies have indicated that p53 plays a crucial role in inducing apoptosis as well as cell cycle checkpoints in human and murine cells following DNA damage primarily through induction of p21 and Bax^32^,^33^,^34^,^35^. p21 regulates cell cycle progression at G1 or G2/M phase. Bax promotes the release of cytochrome c from mitochondria, resulting in the apoptotic caspase cascade. Classical theories hypothesize that cell cycle arrest is a mechanism of self-protection, to enable sufficient time to repair DNA damage. If cells are efficiently repaired, they will reenter the cell cycle. Once the DNA damage is too severe to be repaired, the cells will ultimately die. Our results were consistent with the theory. The carbon ion irradiation caused a serious morphological damage and significant rise in apoptosis in mouse testis. At the mean time, it also induced p53 and deduced p21expression, indicating that the damage of carbon ion irradiation was too severe to be repaired and the radiation induced apoptosis was at least in part in a p53-dependent manner. We examined its involvement in DADS-deduced apoptosis. Unexpectedly, we found that DADS up- regulates p53 and p21 expression in mouse testis exposed to carbon ion irradiation. Previous studies also showed that p53 and p21 were involved in the inhibition of cell cycle progression^36^,^37^,^38^. Therefore, p53 might be involved in the inhibition of cell cycle progression through up regulation of p21 but does not appear to be critical for DADS-deduced apoptosis in mouse testis exposed to carbon ion irradiation.

p73 expression is studied to induce mitochondrial-mediated apoptosis, which is the best known intrinsic apoptotic pathway^39^,^40^. The p73 transcription factors are present in two forms, the full length Tap73 and the N-terminally truncated ∆Np73. In cultured sympathetic neurons, overexpression of ∆Np73 inhibited apoptosis induced by nerve growth factor withdrawal or p53 overexpression^41^. Tap73 had the potential to activate p53 responsive genes and had the ability to induce apoptosis in a p53-independent manner^26^,^27^,^42^,^43^,^44^,^45^. Christine *et al.* showed that Tap73 could, at least when overproduced, activate the transcription of p53-responsive genes and inhibit cell growth in a p53-like manner by inducing apoptosis (programmed cell death)^28^. Our previous research showed that, in the absence of functional p53, family members like ∆Np73 might be important in radiation-induced apoptosis in human cervical cancer cells^26^. Another studies also revealed that a surprising neuroprotective role of ∆Np73 isoform^41^,^46^. Indeed, many *in vitro* and *in vivo* evidences demonstrated that the Tap73 was a pro-apoptotic isoform and ∆Np73 was an anti-apoptotic isoform^27^. However a body of research suggested that ΔNp73 overexpression alone didnot confer any growth advantage to tumour cells^47^,^48^. Thus this observation lead to the assumption that ΔNp73 was not an oncogene, nevertheless with genotoxic stimuli or acquisition of a Ras mutation, high ΔNp73 expression does lead to increased cell survival. The contrary of its function may be explained by the following reason: the ration between the TAp73 and ΔNp73 dictates the cellular response. Furthermore, some studies showed that the ratio between Tap73 and ∆Np73 determined the fate of the cell- survive or die^27^,^44^. Although there is ample evident that the p73 is associated with apoptosis, the ratio between Tap73 and ΔNp73 involved in the protective role of DADS in mouse testis exposed to carbon ion irradiation remains unclear^38^,^49^,^50^. In the study, the ratio between Tap73 and ∆Np73, but not the isoform itself, dictated the cellular response to carbon ion irradiation. Hence, our results showed that DADS pretreatment obviously prevented the irradiation-induced increase in the Tap73/ΔNp73 ratio, indicating that administration with DADS alleviates radiation-induced apoptosis in p73 dependent manner.

Several studies have suggested that DADS might regulate caspase-dependent apoptosis through a mitochondrial-mediated intrinsic pathway^51^,^52^,^53^. To better understand how DADS regulates the mitochondrial apoptosis pathway in mouse testis exposure to carbon ion particles, the relative levels of pro-apoptosis versus anti-apoptosis Bcl-2 family, cytochrome c release and caspase-3 activation were determined in all treatments group at 48 h after irradiation. Noticeably, our data revealed that DADS employing decreased the Bax level and Bax/Bcl-2 ratio. This indicated that the modulation of the levels of anti-apoptotic and pro-apoptotic protein in the mouse testis mediated by DADS probably inhibited irradiation-caused mitochondria-dependent apoptosis. The observation of Yin *et al.* also reported that DADS regulated apoptosis by activating a mitochondria-dependent pathway with the increasing Bax/Bcl-2 ratio^54^. Lee *et al.* obtained similar results that lessened apoptosis by DADS was associated with a reduction in Bax in rats treated with hepatotoxicants^20^. Additionally, we also found that the cytochrome c markedly increased in the mouse testis at 48 h after carbon ion irradiation. However, intraperitoneal injection of DADS on the irradiated mice could suppress the amount of cytochrome c release, which was a critical step to block cell death program. Previouse research reported that efflux cytochrome c from mitochondria to the cytosol was essential for caspase-3 activation and activated downstream cell death pathway^55^,^56^. Moreover, the research from our study found that the activation of caspase-3 was deduced by carbon ion irradiation, and the cleavage of caspase-3 was found in Fig. 6, while daily administration of DADS could obviously induce the expression of caspase-3 and limit its specific cleavage (Fig. 6). The observation of Nagathihalli *et al.* also found that DADS regulated apoptosis through the mitochondrial pathway, as evidenced by the loss of mitochondrial membrane potential and the release of mitochondrial cytochrome c^52^. Interestingly, some studies showed that pretreatment with DADS attenuated EtOH-induced gastric apoptotic changes, carbon tetrachloride-induced hepatic apoptotic changes, and oxidative stress-injured neuronally differentiated PC12 cells, as evidenced by inhibition of cytochrome c release and caspase-3 activation^19^,^20^,^21^.

**Figure 7:**
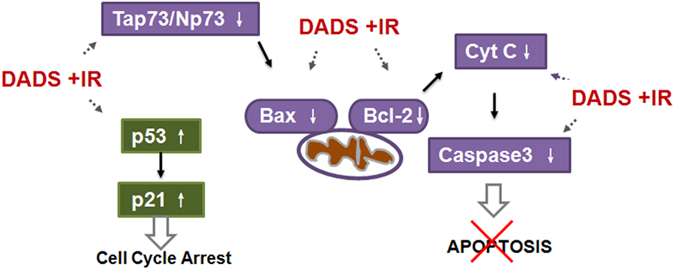
Model of potential targets of DADS and irradiation on apoptosis-inducing mitochondrial pathway in mouse testis. DADS supplementation elevated p53 and p21 expression which regulated DNA repair. At the mean time, administration with DADS prevented Tap73/ΔNp73 expression which regulated apoptosis, and consequently down regulated Bax/Bcl-2 ratio, cytochrome c release and caspase-3 expression in mouse testis exposed to carbon ion irradiation.

Taken together, our results strongly suggest that DADS ameliorates carbon ion irradiation-induced damage in mouse testis by activation of DNA repair via p53/p21 and blocking apoptosis through the ratio of Tap73/ΔNp73 via mitochondrial pathway (Fig. 7). However, this would require further investigation using tumor-bearing animal model and clinical trials to understand the effects of the DADS on the tumor and the normal surrounding tissue.

## Additional Information

**How to cite this article**: Di, C.-x. *et al.* Diallyl disulfide attenuated carbon ion irradiation-induced apoptosis in mouse testis through changing the ratio of Tap73/∆Np73 via mitochondrial pathway. *Sci. Rep.* **5**, 16020; doi: 10.1038/srep16020 (2015).

## Figures and Tables

**Table 1:** Primers used in qRT-PCR

Name	Forward primer sequence	Reverse primer sequence
p53	5′-GCCGACCTATCCTTACCATC-3′	5′- CAGCCGAGCCAGTAATAG-3′
Tap73	5′-GGAGATGGCCCAGACCTCTTCTTCC-3′	5′-CTAGACTTCGAGCAGGAGATGG-3′
Np73	5′-TTGAAGTCCCTTCCAAGCTCGTGGT′	5′-CTTTACGTCGGTGACCCCATGAGAC-3′
β-Actin	5′-ACTGTGTTGGCATAGAGGTCTTTA-3′	5′-CTAGACTTCGAGCAGGAGATGG-3′
